# Targeting degradation of IKZF1 and IKZF3 through modulation of the E3 ligase substrates in the context of cellular therapies for multiple myeloma

**DOI:** 10.1186/s40364-025-00825-8

**Published:** 2025-08-15

**Authors:** David Kegyes, Anamaria Bancos, Adrian Bogdan Tigu, Ioana Rus, Delia Dima, Diana Cenariu, Madalina Nistor, Raluca Munteanu, Diana Gulei, Alina Tanase, Anca Colita, Anca Buzoianu, Cristina Iuga, Mihnea Zdrenghea, Evangelos Terpos, Stefan O. Ciurea, Aaron Ciechanover, Hermann Einsele, Ciprian Tomuleasa

**Affiliations:** 1 Department of Translational Medicine, Medfuture Institute of Medical Research and Life Sciences, Cluj-Napoca, Romania; 2 Department of Hematology, Iuliu Hatieganu University of Medicine and Pharmacy, Cluj-Napoca, Romania; 3 Department of Stem Cell Transplantation, Fundeni Clinical Institute, Bucharest, Romania; 4 Department of Clinical Therapeutics, School of Medicine, National and Kapodistrian University of Athens, Alexandra General Hospital, Athens, Greece; 5 Hematopoietic Stem Cell Transplantation and Cellular Therapy Program, Division of Hematology/Oncology, Department of Medicine, University of California Irvine, Irvine, CA USA; 6 Department of Cell Biology and Cancer Science, Rappaport Faculty of Medicine, Technion- Israel Institute of Technology, Haifa, Israel; 7 Department of Internal Medicine II, Julius Maximilians University, Wurzburg, Germany

**Keywords:** Multiple myeloma, CELMoD, Iberdomide, CC-220, Mezigdomide, CC-92480, Immunotherapies, CAR t-cell therapy, T-cell engager, Autologous hematopoietic stem cell transplantation

## Abstract

Multiple myeloma (MM) is a blood cancer characterized by the clonal evolution of plasma cells. In 2022, there were an estimated 118 000 MM cases and 121 000 deaths worldwide. The treatment landscape of MM has undergone a dramatic transformation in recent decades, shifting from conventional chemotherapy to more targeted approaches. In order to overcome intrinsic and acquired resistance mechanisms that frequently restrict the efficacy of single-agent therapies, drug combination strategies have been developed to simultaneously target multiple pathogenetic pathways. Building on the success of immunomodulatory agents, CRBN E3 ligase modulators (CELMoDs), iberdomide (CC-220) and mezigdomide (CC-92480), have been designed as promising and more selective agents. CELMoDs demonstrate a 10–20 times higher binding capacity and they promote a more profound and rapid breakdown of Ikaros and Aiolos compared to traditional immunomodulatory agents. According to the National Cancer Institute Surveillance Program, the median survival for fit patients is greater than ten years, and the 5-year survival for the general MM patient population in the US approaches 60%. Despite these encouraging numbers, MM is still an incurable disease, and the majority of patients eventually relapse and require additional lines of therapy. Combining CELMoDs with cellular therapies significantly improves the response rate in MM patients. In this paper, based on the literature presented at the Annual Meeting of the American Society of Hematology (ASH), the American Society of Clinical Oncology (ASCO), the International Myeloma Society (IMS), and the European Hematology Association (EHA) in the 2020–2025 timeframe, we explore the rationale and emerging evidence of combining CELMoDs with immunotherapies, and their use as a bridge to transplant or as post-ASCT maintenance therapy in MM.

## Introduction

Multiple myeloma (MM) is a blood cancer characterized by the clonal evolution of plasma cells. In 2022, there were an estimated 118 000 MM cases and 121 000 deaths worldwide [1]. Signs and symptoms of MM vary in a wide range including hypercalcemia, renal dysfunction, anemia and osteolytic bone lesions, collectively summarized as the “CRAB” criteria. Final diagnosis of MM is made based on serum protein electrophoresis, free light chain assays, bone marrow biopsy, imagistics and cytogenetic/genetic tests [2]. A detailed description of the diagnostic steps is, however, out of the scope of this paper.

The treatment landscape of MM has undergone a dramatic transformation in recent decades, shifting from conventional chemotherapy to more targeted approaches. In order to overcome intrinsic and acquired resistance mechanisms that frequently restrict the efficacy of single-agent therapies, drug combination strategies have been developed to simultaneously target multiple pathogenetic pathways. Current gold standard regimens for newly diagnosed (NDMM) or early relapsed/refractory (RRMM) include proteasome inhibitors (PIs) such as bortezomib (Velcade - V), carfilzomib (Kyprolis - K), and ixazomib (Ninlaro - N), alongside immunomodulatory drugs (IMiDs) like lenalidomide (Revlimid - R) and pomalidomide (P), and dexamethasone (d), respectively. Quadruplet therapies, which add monoclonal antibodies (mAbs) such as daratumumab (D), are more and more used as first-line treatment options [3].

The survival of myeloma cells relies, among others, on the suppressive tumor microenvironment and the ability of malignant cells to evade host immune responses. IMiDs modulate the immune system by co-stimulating CD4 + and CD8 + T-cells, suppressing regulatory T cells (Tregs), and inducing anti-tumoral cytokines such as interleukin-2 (IL-2) and interferon gamma (IFNγ). IMiDs alter myeloma-associated macrophage polarization in the bone marrow microenvironment shifting these cells from an immune-evasive M2-like phenotype to an anti-tumoral M1-like profile that further stimulates T-cell activity [4]. They also activate NK-cells and enhance NK-cell-mediated ADCC. In addition, these molecules exhibit direct anti-proliferative, anti-angiogenic, and anti-osteoclastogenic properties [5]. The primary action of these drugs, however, is associated with cereblon (CRBN) activity, a substrate receptor of the E3 ubiquitin ligase complex. CRBN was first described in 2010 of a primary target of thalidomide which later proved to also be responsible for its teratogenicity [6]. Tagging neo-substrate proteins with the CRBN-ubiquitin ligase complex causes their proteasome-mediated degradation. In the case of MM, IMiDs act as molecular glues binding Ikaros (IKZF1) and Aiolos (IKZF3), two B-cell transcription factors, to the CRBN complex, eventually leading to their destruction [7]. The levels of Ikaros and Aiolos are linked to the levels of several oncogenes involved in MM pathogenesis, such as c-MYC and interferon regulatory factor 4 (IRF4) via a positive-feedback loop [8]. Thus, MYC and IRF4 are downregulated upon Ikaros and Aiolos degradation, removing the anti-apoptotic activity of these oncogenes [9].

Building on the success of IMiDs, CRBN E3 ligase modulators (CELMoDs), iberdomide (CC-220) and mezigdomide (CC-92480), have been designed as promising and more selective agents. CELMoDs demonstrate a 10–20 times higher binding capacity and they promote a more profound and rapid breakdown of Ikaros and Aiolos compared to traditional IMiDs [10, 11].

According to the National Cancer Institute Surveillance Program, the median overall survival (OS) for fit patients is greater than ten years, with 5-year OS in the US approaching 60% [12]. Despite these encouraging numbers, MM is still an incurable disease, and the majority of patients eventually relapse and require additional lines of therapy. Therefore, new and more effective drug combinations must be identified. Preclinical data highlight the potential benefits of combining CELMoDs with immunotherapies, such as autologous stem cell transplantation (ASCT), mAbs, antibody-drug conjugates (ADCs), T-cell engagers (TCEs), chimeric antigen receptor (CAR) T-cell therapies, and immune checkpoint inhibitors (ICI) [13, 14]. In this paper, based on our literature search of PubMed and abstracts presented at the Annual Meeting of the American Society of Hematology (ASH), the American Society of Clinical Oncology (ASCO), the International Myeloma Society (IMS), and the European Hematology Association (EHA) in the 2020–2025 timeframe, we explore the rationale and emerging evidence of combining CELMoDs with immunotherapies, and their use as a bridge to transplant or as post-ASCT maintenance.

## Iberdomide

The first in vitro data about iberdomide, formerly CC-220, were published in 2018 while the first clinical reports in 2022, describing the results of CC-220-MM-001 (NCT02773030), a multicohort, open-label, phase 1/2 study [12]. This trial investigated, among others, iberdomide in combination with dexamethasone in RRMM. In the phase 1 cohort, at a median follow-up of 5.8 months the overall response rate (ORR) was 32%, including 1% complete remission (CR), 9% very good partial response (VGPR), and 22% partial response (PR) (*N* = 90) and the best efficacy/safety ratio was reported with the dose of 1.6 mg. Similar results were observed in the phase 2 cohort, with an ORR of 26% (median follow-up 7.7 months, 1% CR, 8% VGPR, and 18% PR). Based on the initial promising clinical outcomes and further preclinical data suggesting high synergism between these agents, CC-220-MM-001 included other cohorts, such as iberdomide, bortezomib with dexamethasone (VId) and iberdomide, carfilzomib with dexamethasone (KId), respectively [15]. The ORRs improved significantly in the triplet regimens, 56% in the VId arm (*N* = 25) and 50% in the KId arm [16, 17]. The first immunotherapy containing iberdomide regimen tested in CC-220-MM-001 was daratumumab (D) on an iberdomide-dexamethasone backbone (DId).

Daratumumab, an anti-CD38 mAb, proved to be highly synergistic with iberdomide in preclinical models regardless of CD38 expression levels [18, 19]. Preliminary data of **DId** reported an ORR of 100% regardless of the presence of high-risk markers, such as TP53 mutations, del(17p), 1qAMP and t(4;14) in case of transplant-ineligible newly-diagnosed MM (NDMM) patients. Also, minimal residual disease (MRD)-negativity rates at a threshold of 10^− 5^ determined by multiparametric flow cytometry (MFC) were impressive across all dose cohorts (10/17, 58.8% at 1 mg; 13/22, 59.1% at 1.3 mg; 7/16, 43.8% at 1.6 mg) [20]. A direct comparison of **DId ***versus ***Id** along in transplant-ineligible NDMM (*N* = 140) has been initiated by the phase 2 GEM-IBERDARAX study (NCT05527340). While CC-220-MM-001 administered intravenous daratumumab, in GEM-IBERDARAX the subcutaneous formulation was used. Daratumumab-containing quadruplets are also increasingly investigated in MM. **DIVd** and **DIKd** are two iberdomide-containing regimens currently in phase 1/2 trials. In the IDEAL study (NCT05392946), NDMM patients receive **DIVd** for 12 cycles followed by an iberdomide monotherapy maintenance for up to 36 cycles.

In case of RRMM, CC-220-MM-001, after a median follow-up of 4.17 months, reported ORR of 46% with VGPR or better in 24% of patients (*N* = 43) receiving **DId.** Median time of response was 4.1 months (95% CI, 4.0–12 months) [21, 22]. In order to determine whether a CELMoD- or a PI-containing triplet would lead to better outcomes in RRMM, the phase 3 EXCALIBER-RRMM (NCT04975997) trial will directly compare **DId** with daratumumab-bortezomib-dexamethasone (**DVd**) [23]. **DIKd** in NCT05896228 will be administered to RRMM patients for 8 cycles followed by 12 months of iberdomide maintenance. ORR, CR rates and adverse events (AEs) are the main outcomes to be determined in both studies.

Isatuximab, recently FDA-approved as an alternative to daratumumab, is used in combination with bortezomib, lenalidomide, and dexamethasone for NDMM. Comparative studies show that isatuximab and daratumumab have similar efficacy and patients resistant to daratumumab typically exhibit poor responses to isatuximab [24]. It remains unknown whether daratumumab or isatuximab is superior when combined with CELMoDs, as no clinical trials have directly addressed this question. The ongoing NCT05558319 trial is comparing isatuximab-VRd with isatuximab-VId in transplant-eligible NDMM. The BOREALIS study (NCT05272826) is recruiting NDMM patients to assess the benefit of adding isatuximab to IVd in cases with persistent MRD after 4 cycles of IVd. Additionally, the phase 3 NCT06216158 trial evaluates post-ASCT maintenance with an iberdomide–isatuximab combination versus iberdomide alone.

Elotuzumab is an anti-SLAM7 mAb approved for treatment of RRMM in combination with pomalidomide and dexamethasone. Resistance to IMiDs, such as pomalidomide, however, requires identification of novel efficient elotuzumab combinations. While treatment with iberdomide increases surface CD38 levels but has no significant effect on SLAMF7 expression [25]. Nevertheless, when combined with elotuzumab, iberdomide enhances immune-mediated killing of MM cell lines, leading to a more pronounced response than pomalidomide [25]. Therefore, two phase 1/2 clinical trials have been initiated to investigate elotuzumab-iberdomide added to dexamethasone (NCT05560399) or the elotuzumab-daratumumab-iberdomide-dexamethasone quadruplet (NCT06785415).

The bispecific antibodies, teclistamab and elranatamab (both CD3-BCMA TCEs); talquetamab (a CD3 and human G-protein coupled receptor family C group 5 member D - GPRC5D targeting TCE) and cevostamab (an anti-CD3 and Fc receptor-homolog 5 - FcRH5 TCE), are currently under investigation in combination with iberdomide in several phase 1/2 clinical trials, including NCT06348108, NCT06215118 (MagnetisMM-30), NCT06465316, and NCT05583617 (PLYCOM). These trials focus on patients with RRMM, aiming to determine efficacy and safety of these novel drugs in heavily pretreated populations. Notably, dexamethasone was included only in the talquetamab-iberdomide trial. As of now, no results have been reported. Preclinical data, however, show that combining bispecifics, such as forimtamig, an anti-GPRC5D and CD3 antibody, with CELMoDs leads to high synergy and restores sensitivity even in previously forimtamig-resistant mice models. Also, iberdomide might decrease the incidence of cytokine release syndrome (CRS) by modulating cytokine expression and lowering levels of IL-2, IL-6 or IL-8 [26].

Belantamab mafodotin is a BCMA-directed antibody-drug conjugate (ADC) approved for the treatment of RRMM. NCT06232044 is a phase 1/2 trial that is evaluating the potential synergistic effects of combining belantamab mafodotin with iberdomide, and dexamethasone.

CAR T-cell therapies are among the most effective salvage therapies now on the market, despite not being generally accessible yet. The long-term outcomes of these adoptive cellular therapies are significantly limited by resistance mechanisms, including antigen escape, T-cell depletion, and the immunosuppressive tumor microenvironment. BCMA-targeting CARs, such as idecabtagene autoleucel and ciltacabtagene autoleucel are already FDA-approved agents. In vitro, coculturing iberdomide with anti-BCMA CAR T-cells led to increased viability (80% *versus* 30%, *P* < 0.0001), increased the expression of activation markers HLADR and CD69 (*P* < 0.05) and increased antigen specific production of cytokines by CAR T-cells (*P* < 0.01). Also, cell killing efficacy of CAR T-cells was increased by 46% compared to control (*P* < 0.001) [27]. According to preclinical data, we are awaiting the initiation of clinical trials combining CELMoDs with BCMA-targeted CAR T-cells. Currently, only post-ide-cel maintenance therapies are being assessed in randomized clinical trials. In the phase 2 NCT06179888 iberdomide maintenance will be administered until disease progression or unacceptable toxicities. Iberdomide will be initiated 80–110 days after administration of idecabtagene. Given the relatively short PFS in patients with RRMM, the study will enroll patients regardless of initial response to ide-cel, also patients in CR with negative MRD-tests. NCT06518551 is another phase 1/2 trial investigating the effects of the elotuzumab-iberdomide-dexamethasone triplet administered until disease progression within 90 days of CAR T-cell infusion to patients that achieved at least partial response following CAR T-cell therapy. The only CAR T-cell trial investigating the co-administration of iberdomide with CAR T-cells is ​ NCT06121843 which compares safety and efficacy of arlocabtagene autoleucel (BMS-986393, a GPRC5D-targeting agent) in combination with alnuctamab, iberdomide or mezigdomide. First preliminary results might be published in the next 2–3 years.

The current standard of care for post-ASCT maintenance in MM is primarily based on the use of lenalidomide. IMiDs, by suppressing pro-tumoral helper T-cell subtypes such as Th17 and Th22, improve post-ASCT outcomes when used as part of induction therapy combinations [47]. Despite its benefits, lenalidomide maintenance is associated with risks such as second primary malignancies and hematologic toxicities, leading to exploration of alternative strategies. With a good safety profile and superior response at six months, iberdomide proved, based on preliminary data, a novel and successful post-ASCT maintenance therapy. The EMN26 study (NCT04564703) is the first multicohort, phase 2 trial evaluating iberdomide as post-ASCT maintenance. Three dose cohorts were enrolled, 0.75, 1.0, or 1.3 mg on days 1–21 of each 28-day cycle. Subjects who met the IMWG criteria for at least a partial response (PR) following ASCT and who were diagnosed within 12 months and 120 days of the last ASCT were enrolled. They received a PI and IMiD +- daratumumab containing induction, 19% received double ASCT, and 7% underwent post-ASCT consolidation. Since the 0.75 mg iberdomide arm was initiated later, no data has been yet reported. In total 69 patients (34 in the 1 mg arm, 35 in the 1.3 mg arm) received 6 or more cycles of iberdomide. In terms of efficacy, 48% (90% CI, 32–65%) of patients treated with 1.0 mg iberdomide exhibited an improvement in response (51% sCR after 6 months compared to 17% at study enrollment, 9% *versus* 11% CR, 37% *versus* 69% VGPR, 3% *versus* 3% PR). Efficacy was similar in the 1.3 mg cohort with 45% (90% CI, 29–62%) ORR (47% *versus* 15% sCR, 3% *versus* 12% CR, 44% *versus* 59% VGPR, 6% *versus* 15% PR). PFS for the 1.0 and 1.3 mg cohorts at 6 months was 97% and 94%, respectively [48]. No MRD data have been published yet. Indirect comparison of response improvements with iberdomide compared to lenalidomide suggests superiority of iberdomide (48% *versus* 26% at 6 months based on the EMN02 study). However, a direct comparison is required, and the phase 3 EXCALIBER (NCT05827016) is currently recruiting subjects to evaluate the efficacy of iberdomide *versus* lenalidomide maintenance until disease progression. In terms of safety, neutropenia (46% in the 1.3 mg cohort and 21% in the 1.0 mg cohort), infections (14% and 3%), and fatigue (14% and 12%) were the most frequent grade ≥3 AEs during iberdomide maintenance in EMN26. AEs were managed with dose reductions in 31% of subjects in the 1.3 mg group and 18% in the 1.0 mg cohort. Three patients in the 1.0 mg (1 due to AEs, two PD) and four patients in the 1.3 mg arm discontinued iberdomide (2 due to AEs, one PD, and one death of unknown cause) [48]. Sequential therapy with iberdomide following at least 6 months of lenalidomide maintenance or following lenalidomide maintenance and second, salvage ASCT is also tested in the phase 2 NCT05354557 trial. Currently available studies demonstrate the efficacy of iberdomide monotherapy. It is unclear, however, whether iberdomide-containing doublets or triplets are preferable maintenance therapies. The phase 2 IBEX study (NCT06107738) combines daratumumab and iberdomide in patients MRD-positive 90–150 days after ASCT by ClonoSEQ assay with a sensitivity of 10^− 5^. The doublet will be administered for 26 cycles. A dose of 1 mg of iberdomide was chosen for the first three cycles, with a dose increase to 1.3 mg after the fourth cycle if tolerated. Efficacy endpoints of the trial are MRD-negativity after 12 months of treatment, the rate of sustained MRD-negativity after 24 months, PFS, OS and rate on improvement in IMWG response category. NCT06216158 is currently testing the isatuximab-iberdomide doublet given for 39 cycles in MRD + patients post-ASCT patients. The COMMANDER trial (NCT05434689) is evaluating the MRD (determined by ClonoSEQ 60–120 days post-ASCT) conversion rate of 6 cycles of iberdomide, daratumumab and dexamethasone or 6 cycles of iberdomide, carfilzomib, daratumumab and dexamethasone (Table 1).

**Table 1:** Iberdomide-based trials

Drug/Combination	NCT ID	Phase	*N*	Patient Population	Efficacy data	Adverse events
**DId ** *versus * **Id**	NCT02773030 (CC-220-MM-001)	1/2	55	transplant-ineligible NDMM	ORR: 100%; MRD-negativity at 10^− 5^: 13/22 pts (59.1%);	Grade 3 or higher neutropenia, infections in 59%), discontinuation due to AEs in 2%
**DId ** *versus * **Id**	NCT02773030 (CC-220-MM-001)	1/2	43	RRMM	ORR: 46%, ≥VGPR: 24%, CR: 8%, Median duration of response: 35.7 months (median follow-up 4.17 months)	Grade 3 or higher neutropenia in 67% and anemia in 21%
**DId** *versus* **Id** (data only for the Id arm available)	NCT05527340 (GEM-IBERDARAX)	2	18	transplant-ineligible NDMM	ORR: 82% (14/17), CR: 35%,	Grade 3 or higher neutropenia in 78%, infections 72%
**DId ** *versus * **DVd**	NCT04975997 (EXCALIBER-RRMM)	3	864	RRMM	No data published	
**DVId**	NCT05392946 (IDEAL)	1/2	49	NDMM	No data published	
**DKId**	NCT05896228	2	30	RRMM	No data published	
Isatuximab-**VId**	NCT05272826 (BOREALIS)	2	75	transplant-ineligible NDMM	No data published	
Isatuximab-**VId**	NCT05558319	3	480	transplant-eligible NDMM	No data published	
Elotuzumab-**Id**	NCT05560399 (GCO 22 − 0016)	1/2	25	RRMM	No data published	
Elotuzumab-**DId**	NCT06785415	1/2	37	RRMM	No data published	
Elranatamab-**I**	NCT06215118 (MagnetisMM-30)	1	87	RRMM	No data published	
Belantamab Mafodotin-**I +- d**	NCT06232044	1/2	88	RRMM	No data published	
Talquetamab-**Id**	NCT06348108	1	38	RRMM	No data published	
Teclistamab-**I**	NCT06465316	1	26	RRMM	No data published	
Cevostamab-**I**	NCT05583617 (PLYCOM)	1/2	200	high-risk NDMM post-ASCT	No data published	
**I** post-ASCT	NCT05177536	2	42	NDMM post-ASCT	No data published	
**I** post-ASCT	NCT05354557	2	86	NDMM post-ASCT	No data published	
**I** post-ASCT	NCT04564703 (EMN26)	2	160	NDMM post-ASCT	48% ORR, 97% PFS at 6 months	46% neutropenia, 14% infections, 14% fatigue
**I ***versus ***R** post-ASCT	NCT05827016 (EXCALIBER)	3	1216	NDMM post-ASCT	No data published	
**ID** post-ASCT	NCT06107738 (IBEX)	2	60	MRD + post-ASCT	No data published	
Isatuximab-**I** post-ASCT	NCT06216158	3	411	NDMM post-ASCT	No data published	
**DId** or **DKId** post-ASCT	NCT05434689 (COMMANDER)	1/2	80	NDMM post-ASCT MRD+	No data published	
**I** post-**CAR T** (Ide-Cel)	NCT06179888 (NCI-2023-10541)	2	78	RRMM	No data published	
Elotuzumab-**Id** post-**CAR T** (Ide-Cel)	NCT06518551	1/2	49	RRMM	No data published	
**CAR T** (Arlo-Cel) - BMS-0986393) + **I**	NCT06121843 (CA088-1005)	1	111	RRMM	No data published	

Abbreviations. Arlo-Cel – arlocabtagene autoleucel; ASCT – autologous stem cell transplantation; D – daratumumab; d – dexamethasone; K – carfilzomib; I – iberdomide; Ide-Cel – idecabtabtagene autoleucel; N – ixazomib; NDMM – newly-diagnosed MM; R – lenalidomide; V – bortezomib

## Mezigdomide

Mezigdomide, like iberdomide, has been developed by Bristol-Myers Squibb. In preclinical models of MM mezigdomide has higher affinity to CRBN compared to iberdomide and IMiDs [28–30]. Mezigdomide demonstrated antiproliferative and proapoptotic properties across MM cell lines, including those exhibiting resistance to lenalidomide and pomalidomide, as well as primary myeloma cells with diminished or mutated CRBN expressions. The precise mechanisms that lead to the enhanced efficacy, however, are not yet clear.

Immunologic studies revealed that patients with RRMM show significantly lower NK-cell, CD4 + and CD8 + T-cell number, all these findings being suggestive of an immune system dysregulation and exhaustion. CELMoDs, and mostly mezigdomide, seem to improve CD8 + T-cell immunosurveillance by MHC class I-mediated, modified antigen presentation of neo-substrates to be degraded by CRBN E3 ubiquitin ligase [34]. Zhao et al. reported that in vitro culturing of mezigdomide with activated T-cells induced significant reduction of exhaustion associated markers [35]. A biomarker analysis by Stong et al. revealed that mezigdomide, independent of prior lines of therapy, induced a gradual immunostimulation, increasing the levels of CD4 + proliferative T-cells, CD4 + effector memory T-cells and also the number of activated CD4 + T-cells [31]. Oekelen et al. reported a significant expansion of the total NK cell population (*P* < 0.01) and a shift of both CD4 + and CD8 + T-cell populations to an effector phenotype [36].

**Fig. 1:**
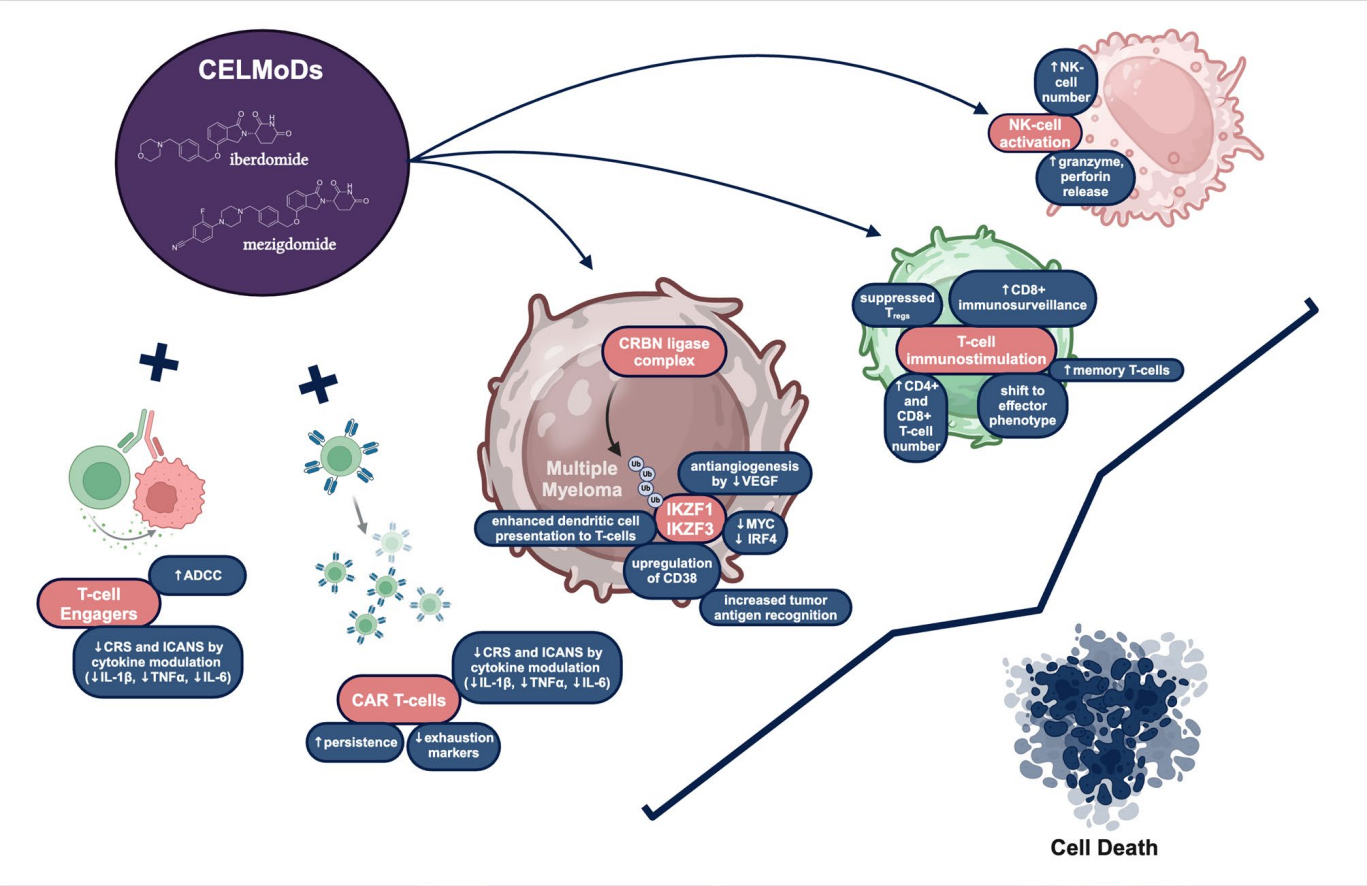
Immunological effects of mezigdomide

Additionally, mezigdomide exhibits a notable increase in the levels of IL-2 and IFN-γ and up to three times greater inhibition of IL-6, IL-1β, and TNFα than iberdomide, pomalidomide, or lenalidomide (Fig. 1). Its inhibitory effects on cytokine release by macrophages, monocytes, and other peripheral blood mononuclear cells suggests that mezigdomide might significantly reduce the incidence of CRS and immune cell therapy associated neurotoxicity syndrome (ICANS) following treatment with T-cell engagers or CAR T-cell therapies [45]. Preclinical studies show a high synergy between the BCMA-targeting TCE, alnuctamab and mezigdomide. Mezigdomide reversed T-cell exhaustion and led to T-cell activation in ex vivo MM and in vivo xenograft models [46]. Moreover, Meermeier et al. demonstrated that high tumor burden limits T-cell activation and is linked to primary resistance to T-cell engagers. Mezigdomide seems to mitigate this T-cell exhaustion and overcome high tumor burden associated resistance in vivo, in mice xenografts [37] (Fig. 1).

The expression levels of checkpoint inhibitors seem to be accurate prognostic biomarkers of response to mezigdomide. Oekelen et al. reported a significant upregulation of programmed death-1 (PD1) and T cell immunoreceptor with Ig and ITIM domains (TIGIT) on the surface of T- and NK-cells in mezigdomide-resistant patient samples. The upregulation of these immune checkpoints was dose-dependent and markedly elevated at higher mezigdomide doses (*P* < 0.05) [36].

Adding dexamethasone to mezigdomide does not significantly affect immunostimulatory effects in vitro [31]. In xenograft models, however, mezigdomide showed considerable synergism when combined with dexamethasone, resulting in marked inhibition of tumor growth (84%) in comparison with either agent alone (34% for mezigdomide and 20% for dexamethasone) [30, 38]. Mezigdomide and dexamethasone were administered to patients with RRMM in the CC-92,480-MM-001 (NCT03374085) trial, a phase 1/2, open-label, multicenter study that included a dose-escalation (*N* = 77) and a dose-expansion cohort (*N* = 101). In phase 1 the ORR was 25% at a median follow-up of 6.3 months, with 1% obtaining a CR, 12% a VGPR, and 12% a PR. The suggested dose of mezigdomide, combined with dexamethasone, was established at 1.0 mg once daily for 21 days. In phase 2 part, all patients were triple-class refractory and 30% had received anti-BCMA therapy. The ORR was 41% at a median follow-up of 7.5 months, with 2% obtaining sCR, 3% CR, and 20% VGPR. Additionally, subgroups with plasmacytoma (30%) and prior anti-BCMA medication (50%), showed favorable ORR outcomes. The promise of mezigdomide plus dexamethasone in RRMM, including patients refractory to anti-BCMA therapies, is highlighted by the median PFS of 5.4 months noted among patients who had previously received anti-BCMA therapy. However, the high incidence of infections (35%), anemia (36%), grade 3 or higher neutropenia (76%), and febrile neutropenia (15%) must be taken in consideration.

Proteasome activity is necessary for mezigdomide-induced degradation of Ikaros and Aiolos. The combination of bortezomib and mezigdomide, however, results in significantly enhanced cytotoxicity against MM cells in vitro and significant tumor regression in vivo [27]. The paradoxical synergism between the IMiDs/CELMoDs and PIs is attributed to additional non-ubiquitin-mediated effects of targeting CRBN. These include the destabilization and accumulation of misfolded transmembrane proteins in myeloma cells that activates several cell death mechanisms [39]. The CC-92480-MM-002 trial (NCT03989414) confirmed the clinical efficacy of both mezigdomide-bortezomib-dexamethasone and mezigdomide-carfilzomib-dexamethasone with an ORR of 84.2% (32/38) and 85.2% (23/27). Median duration of response has not been reached in the bortezomib arm and it was 11.9 months (no 95% CI reported) in the carfilzomib arm after a follow-up of 10.8–13.2 months [40]. These findings highlight the need of novel clinical trials investigating mezigdomide-containing triplet/quadruplet regimens. The SUCCESSOR-1 trial (NCT05519085) is currently recruiting subjects to directly compare mezigdomide-bortezomib-dexamethasone to pomalidomide-bortezomib-dexamethasone in RRMM [41].

It has also been previously shown that Ikaros and Aiolos suppress CD38 expression [42]. Mezigdomide pretreatment, probably by degradation of these molecules, increases CD38 cell surface expression, which enhances the anti-myeloma effects of daratumumab and isatuximab [38]. These effects are dose-dependent and more pronounced with longer mezigdomide exposure (21 days and 14 days *versus* 7 days/cycle) [43]. The CC-92480-MM-002 (NCT03989414) trial proved the safety and efficacy of daratumumab, isatuximab or elotuzumab on a mezigdomide-dexamethasone backbone. Results from the daratumumab cohort (*N* = 56) demonstrated an ORR of 75%, comprising of 4% sCR, 14% CR, 29% VGPR, and 29% PR. Predominantly hematological grade 3 or higher AEs have been reported, mostly neutropenia (54%), anemia (11%) and thrombocytopenia (7%). Patients in the elotuzumab cohort (*N* = 20) achieved an ORR of 45% with 5% sCR, 5% VGPR, and 35% PR. Grade 3 or higher neutropenia has been observed in 40% of patients, anemia in 20%, thrombocytopenia in 10%. Compared to the daratumumab arm with only 20%, infection rates were higher with elotuzumab (35%) [44]. Median duration of response and PFS are not reached. Data about the isatuximab-containing arm are not yet reported. NCT05981209 is a novel phase 1 also investigating the elotuzumab-mezigdomide-dexamethasone triplet administered until disease progression in patients with prior exposure to BCMA-targeted therapies.

NCT06163898 is the first clinical study investigating the bispecific antibody alnuctamab with mezigdomide-dexamethasone in triple-refractory RRMM. CAR T-cells combined with mezigdomide are a novel hot topic and more trials will be initiated in the future. As previously described with iberdomide too, arlocabtagene autoleucel is tested in combination with mezigdomide in NCT06121843. A phase 1 study evaluating mezigdomide maintenance initiated between 30 and 90 days post-idecabtagene autoleucel administration is ongoing. We are awaiting the first results of these trials.

Favorable responses to mezigdomide have been observed in patients with TP53 mutations, del17p, 1qAmp, t4:14. Non-responders showed high baseline EZH2 expression which was associated with an inferior PFS (*P* < 0.05) [31]. Therefore, the EZH2 inhibitor tazemetostat combined with mezigdomide has been investigated in the CA057-003 phase 1/2 trial (NCT05372354). Preliminary data report an ORR of 54% with a median follow-up 4.1 months. Interestingly, combining mezigdomide with trametinib, a MEK-inhibitor which might indirectly affect expression levels of EZH2, led to a significantly higher ORR of 92%. Further follow-up is needed in order to investigate long-term efficacy of these combinations [32, 33] (Table 2).

**Table 2:** Mezigdomide-based trials

Drug/Combination	NCT ID	Phase	*N*	Patient Population	Efficacy data	Adverse events
**DMd**	NCT03989414 (CC-92480-MM-002)	1/2	56	RRMM	75% ORR (4% sCR, 14% CR, 29% VGPR, 29% PR)	grade 3 or higher neutropenia in 54%, anemia in 11% and thrombocytopenia in 7%
**Isatuximab-Md**	NCT03989414 (CC-92480-MM-002)	1/2	ND	RRMM	No data published	
**Elotuzumab-Md**	NCT03989414 (CC-92480-MM-002)	1/2	20	RRMM	45% ORR (5% sCR, 5% VGPR, 35% PR)	grade 3 or higher neutropenia in 40%, anemia in 20% and thrombocytopenia in 10%
**Elotuzumab-Md**	NCT05981209 (NCI-2023-0551)	1	27	RRMM	No data published	
**Elranatamab-M**	NCT06645678 (MELT-MM)	1/2	75	RRMM	No data published	
**Alnuctamab-Md**	NCT06163898 (CA058-002)	1	156	RRMM	No data published	
**Arlo-cel-M**	NCT06121843 (CA088-1005)	1	111	RRMM	No data published	
**M** **post-ide-cel**	NCT06048250 (NCI-2023-06771)	1	15	RRMM	No data published	

Abbreviations. Arlo-cel – arlocabtagene autoleucel (BMS-0986393); ASCT – autologous stem cell transplantation; D – daratumumab; Ide-cel – idecabtagene vicleucel; M – mezigdomide; d – dexamethasone; NDMM – newly-diagnosed MM; RRMM – relapsed/refractory MM;

## Conclusion

CELMoDs proved to be active and safe both in monotherapy [49]. Also, several iberdomide- and mezigdomide-based combinations demonstrated significant efficacy, even in hard-to-treat patient populations such as those heavily pretreated, previously exposed to anti-BCMA therapies, and those with high-risk cytogenetic profiles. Overall, CELMoDs exhibit a manageable safety profile, consistent with the known class-related toxicities of IMiDs—most notably cytopenias and infections.

Resistance mechanisms, however, are known to occur with both IMiDs and CELMoDs. To address this challenge, two key strategies can be pursued: the development of next-generation CELMoDs and the exploration of novel CELMoD-based combinations. Regarding the former, CC-885, for instance, has shown activity against MM cell lines and in vivo mouse models [50]. Phase 1 safety trials in RRMM have also been initiated for avadomide (CC-122) and CFT7455. Other emerging CELMoDs—such as CC-99282, eragidomide (CC-90009), BTX-1188, CC-91633, CC-647, and CC-3060—have yet to be evaluated in MM but may demonstrate promising cytotoxic potential in future studies [29].

Regarding the latter strategy, our paper highlights the existing gap in clinical evidence related to CELMoD combinations. Immunotherapies—especially bispecific antibodies and CAR T-cell therapies—are becoming increasingly important for patients who are refractory to IMiDs, proteasome inhibitors, and anti-CD38 monoclonal antibodies. However, their widespread use is limited by several factors, including patient-related aspects such as frailty and performance status, limited drug availability, high costs, potential AEs, and emerging resistance mechanisms. In vitro studies show that combining CELMoDs with immunotherapies enhances immune activation and restores tumor cell targeting, even in immunosuppressive environments or in the presence of antigen escape—a common resistance mechanism to immunotherapy. Some clinical data on these combinations—though mostly preliminary—are already available in RRMM. Additionally, more and more trials are being initiated to investigate their use in NDMM or even in high-risk smoldering MM (SMM) cases. One such example is the isatuximab–iberdomide combination being evaluated in the MODIFY trial (NCT06762769) for SMM.

In conclusion, advancing our understanding of CELMoDs will require a sustained commitment to both basic and translational research. The encouraging data available so far not only highlight the therapeutic potential of these agents but also call for a collective effort from researchers, academic institutions, and the pharmaceutical industry to further explore and refine their use. Continued investment in this field holds the promise of offering a cure or at least improved survival and quality of life for patients facing resistant disease.

## Data Availability

No datasets were generated or analysed during the current study.
